# Parental Perceptions of Quality of Life in Children on Long-Term Ventilation at Home as Compared to Enterostomy Tubes

**DOI:** 10.1371/journal.pone.0149999

**Published:** 2016-02-25

**Authors:** Brahim Redouane, Eyal Cohen, Derek Stephens, Krista Keilty, Marialena Mouzaki, Unni Narayanan, Theo Moraes, Reshma Amin

**Affiliations:** 1 Division of Respiratory Medicine, The Hospital for Sick Children, Toronto, Canada; 2 Department of Pediatrics, University of Toronto, Toronto, Canada; 3 Division of Pediatric Medicine, The Hospital for Sick Children, Toronto, Canada; 4 Department of Child Health and Evaluative Sciences, The Hospital for Sick Children, Toronto, Canada; 5 Department of Biostatistics, Design and Analysis, The Hospital for Sick Children, Toronto, Canada; 6 Centre for Innovation and Excellence in Child and Family Centered Care, The Hospital for Sick Children, Toronto, Canada; 7 Division of Gastroenterology, The Hospital for Sick Children, Toronto, Canada; 8 Department of Orthopedic Surgery, The Hospital for Sick Children, Toronto, Canada; 9 Department of Pediatrics, The Hospital for Sick Children, Toronto, Canada; Iranian Institute for Health Sciences Research, ACECR, ISLAMIC REPUBLIC OF IRAN

## Abstract

**Objective:**

Health related quality of life (HRQL) of children using medical technology at home is largely unknown. Our aim was to examine the HRQL in children on long-term ventilation at home (LTHV) in comparison to a cohort using an enterostomy tube.

**Study Design:**

Participants were divided into three groups: 1) LTHV without an enterostomy tube (LTHV cohort); 2) Enterostomy tube (GT cohort); 3) LTHV with an enterostomy tube (LTHV+GT cohort). Caregivers of children ≥ 5 years and followed at SickKids, Toronto, Canada, completed three questionnaires: Health Utilities Index 2/3 (HUI2/3), Caregiver Priorities Caregiver Health Index (CPCHILD), and the Paediatric Quality of Life Inventory (PedsQL). The primary outcome was the difference in utility (HUI2/3) scores between the cohorts.

**Results:**

One hundred and nineteen children were enrolled; 47 in the LTHV cohort, 44 in the GT cohort, and 28 in the LTHV+GT cohort. In univariate analysis, HUI2 mean (SE) scores were lowest for the GT cohort, 0.4 (0.04) followed by the LTHV+GT, 0.42 (0.05) and then the LTHV cohort, 0.7 (0.04), p = 0.001. A similar trend was seen for the HUI3 mean (SE) scores: GT cohort, 0.1 (0.06), followed by the LTHV +GT cohort, 0.2 (0.08) and then the LTHV cohort, 0.5 (0.06), p = 0.0001. Technology cohort, nursing hours and the severity of health care needs predicted HRQL as measured by the HUI2/3.

**Conclusion:**

The HRQL of these children is low. Children on LTHV had higher HRQL than children using enterostomy tubes. Further work is needed to identify modifiable factors that can improve HRQL.

## Introduction

There have been a number of regional and national surveys from around the world demonstrating the increasing use of medical technology at home in children [1,2,3,4,5,6,7,8,9,10,11,12,13,14,15,16,17,18,19,20]. The growth of this population is due to advances in medical care and assistive technologies partnered with an increasing number of decisions being made to institute medical care in children who would have been previously palliated **[21,22]**. Despite the changing paradigm of pediatric chronic care delivery, little is known about the health related quality of life (HRQL) of this burgeoning population.

HRQL can be defined as the child’s physical, emotional and social well-being as reported by the child or proxy, such as a parent [23]. The evaluation of HRQL as a patient-reported outcome is integral to the iterative process of evaluating the management trajectory of these children that has extended past our current knowledge in recent years. Most long-term ventilation at home (LTHV) studies reporting on HRQL have focused on children with specific underlying diagnoses which does not account for the heterogeneity of this population including many rare conditions. However, the combination of diverse (and often rare) underlying conditions, and similar consequences (e.g. functional impairment, reliance on trained caregivers, etc.) suggests that it may be very appropriate to focus on technology assistance as a target of HRQL studies [24]. Long-term home ventilation (LTHV) and enterostomy tubes are two of the most commonly prescribed technologies that support vital functions in children. Comparing these two cohorts of children can provide further insight into the effect of the particular technology on HRQL. Our aim was to examine parental perceptions’ of HRQL using validated inventories in children using LTHV in comparison to a cohort with enterostomy tubes. We hypothesized that children receiving LTHV will have a lower HRQL than a comparator cohort of children with enterostomy tubes given the intense technology dependency of children reliant on ventilators.

## Methods

This was a single center, cross-sectional study conducted in children requiring LTHV and/ or enterostomy tubes. Our tertiary center has the largest LTHV program in the country and services all children in our catchment area. Additionally, our tertiary institution inserts all of the enterostomy tubes in our catchment area. Therefore, all of these children would be followed in one of the above mentioned clinics. This study was approved by the institution’s Research Ethics Board (REB #1000028025). Children followed in Respiratory Medicine, Complex Paediatrics, Neurology or Gastroenterology/Nutrition Clinics at SickKids, Toronto, Canada were eligible. Data collection was performed between January 2011 and September 2013, inclusive. Written consent was obtained from the parental caregivers of all patients. The consent process was approved by the Research Ethics Board at Sickkids. Questionnaires were completed at one time point.

The LTHV cohort included children with chronic respiratory failure receiving ventilation via a tracheostomy or face mask at home. The enterostomy tube cohort (GT) included children with an enterostomy tube. Inclusion criteria were as follows: 1) parental fluency in English; 2) children between the ages of 5–18 years of age; 3) using LTHV or an enterostomy tube for ≥3 months at home; and 4) medical stability. Exclusion criteria included the use of antibiotics in the previous two weeks and/or evidence of an intercurrent illness at the clinic visit. Participants were divided into three cohorts: 1) LTHV but no enterostomy tube (LTHV); 2) LTHV and an enterostomy tube (LTHV+GT), 3) enterostomy tube only (GT).

The following demographic information was collected: first language at home, ethnicity, occupational status of parents, highest level of education of parents, family income, type of housing, number of siblings, patient’s medical history including primary diagnoses, co-morbidities, number of medications, amount of nursing support, and type of ventilation technology at home (noninvasive positive pressure ventilation (NiPPV) or invasive via tracheostomy). The Children with Special Health Care Needs (CSHCN) screener was also used. The CSHCN screener is a consequences-based five-item tool specifically designed to characterize children with health care needs [25,26,27].The number of consequences reported on the screener reflects more severe needs and has been previously correlated with various negative outcomes on the family unit [22].

The patients were consecutively recruited and informed consent was obtained during regularly scheduled clinic visits to avoid bias. The sample size was a convenience sample of our local clinic population.

The primary study outcome was HRQL as measured by the HUI2 and HUI3, parent-proxy versions. The HUI2/3 is a multi-attribute, preference-based measure of HRQL that generates utility scores for single attributes as well as for a multi-attribute composite **[28,29]**. Utility scores for the HUI2/3 are on a scale of 0 (dead) to 1 (perfect health) and negative scores are considered worse than being dead. The HUI2/3 is validated down to 5 years of age **[28,30]**.

Secondary outcome measures included parent-proxy measures of HRQL using the CPCHILD, and the PedsQL. The CPCHILD, first developed for children with cerebral palsy, is a 36 item instrument validated as a proxy measure of functional and health status, caregiver burden and HRQL in children [31]. Standardized scores (0–100) are reported for each domain and in total. The PedsQL is a generic, 23-item multidimensional instrument **[32,33]**. Standardized scores (0–100) are reported for each domain and in total.

Descriptive statistics was used to characterize the study population. Univariate analysis was then performed for HUI2, HUI3, CPCHILD, Peds QL overall scores and the following variables: age, sex, group, ethnicity, number of nursing caregiver hours, number of siblings, number of days admitted to hospital in the last year, CSHCN Screener score, family income, and female caregiver’s highest level of education. Variables with p values <0.2 in the univariate analysis were forced into the regression model. Four separate regression models were generated for each of the HUI2, HUI3, CPCHILD and PedsQL overall scores. Multiple regression modelling was used to adjust for the variables which may influence the outcome (ie adjust for confounding). Statistical Analysis Systems software version 9.2 (SAS Institute, Inc., Cary, NC) was used to conduct all analyses. The significance level was set at 0.05.

## Results

### Study Participants

During the study period, 153 families were screened and eligible for enrolment. One hundred and nineteen (78%) agreed to participate. Out of the 34 families that were not enrolled, 26 did not want to participate, 3 were too overwhelmed, 3 had a language barrier, 1 family had a fear of being identified, and 1 family did not like questionnaires. There were no significant differences in the baseline characteristics of study participants and those that declined participation.

### Descriptive and Outcome Data

Forty-seven patients were enrolled in the LTHV cohort, 44 in the GT cohort, and 28 enrolled in the LTHV+GT cohort. Ten (14%) of the children in the LTHV and the LTHV +GT cohorts were invasively ventilated via a tracheostomy; 2 in the LTHV cohort. There was no missing data. Table 1 demonstrates the baseline demographic information of the three study cohorts. The mean age, number of nursing hours/week, number of days admitted to hospital in the last year, and CSHCN Screener score were significantly different between the cohorts (see Table 1). The LTHV+GT cohort had the longest time on technology, mean (SD) of 7.3 years. The GT cohort had a significantly higher number of days in hospital in the last year with mean (SD) of 13.7 (3.6) days. Seventy-one percent of the LTHV+GT and 48% of the GT received more than 5 hrs/wk of nursing support compared to only 9% of children in the LTHV cohort (P = 0.0001). The mean (SD) CSHCN Screener score (out of total maximum score of 14) was highest in the GT cohort; a higher score is consistent with higher functional needs.

**Table 1:** Baseline Demographics of the Three Study Cohorts: Enterostomy tube (GT), Long-term home ventilation and enterostomy tube (LTHV+GT) and Long-term home ventilation (LTHV).

	Subcategory	GT n = 44	LTHV+GT N = 28	LTHV n = 47	P-Value
Age (yrs)*		10.6 (0.6)	12.7 (0.7)	13.4 (0.5)	0.0011
**Gender (% male)**		20 (45%)	16 (57%)	25 (53%)	0.5911
Time on Technology (yrs)*		6.3 (0.5)	7.3 (0.8)	5.6 (0.4)	0.0958
Number of Days Admitted to Hospital in Last Year*		13.7 (3.6)	5.6 (3.0)	0.9 (0.4)	0.0019
**Nursing Hours Support (%):**					0.0001
	<5 hrs/wk	52	39	91	
	≥5 hrs/wk	48	71	9	
CSHCN Screener Score* **(out of 14)**		12.0 (0.5)	11.8 (0.5)	9.0 (0.7)	0.0002
**Ethnicity (%)Caucasian**		25 (57%)	17 (61%)	31(66%)	0.6681
**Female Caregiver’s Highest level of Education (%):**					0.2044
	High School	82	71	64	
	Post-Secondary	18	29	34	
**Household Income (%)**					0.6328
	Less than or equal to $50 000	52	54	62	
	Over $50 000	43	29	36	
Number of Siblings*		1.6 (0.2)	1.4 (0.3)	1.3 (0.2)	0.5672

*Results expressed as means (SE) unless indicated to be percentages

Central nervous system (CNS) disorders account for the majority of primary diagnoses across all cohorts followed by the musculoskeletal (MSK) category (see Table 2). The majority of the primary diagnoses for the GT cohort were CNS in origin, n = 23/44 (53%) and MSK for the majority of the LTHV cohort, n = 32/75 (43%).

**Table 2:** The Primary Medical Diagnosis by Technology Cohort. The Cells Indicate the Frequency of the Primary Medical Diagnosis by Technology Cohort.

	Major Subgroup	GT	LTHV+GT	LTHV
**Central nervous system N = 41 (34%)**	Birth injury/cerebral palsy	10	3	0
	Acquired or congenital central hypoventilation syndrome	1	3	4
	Other central causes	12	3	5
	Total	23	9	9
**Musculoskeletal N = 33 (33%)**	Muscular dystrophy	0	1	5
	Spinal muscular atrophy	0	4	5
	Other myopathy	1	4	8
	Other musculoskeletal	0	2	3
	Total	1	11	21
**Respiratory N = 18 (15%)**	Upper airway obstruction/Obesity Syndrome	0	0	6
	Cystic Fibrosis	8	0	0
	Prematurity/ Chronic lung disease	0	0	2
	Other respiratory	0	0	2
	Total	8	0	10
**Genetic/Metabolic N = 21 (18%)**	Trisomy 21	1	2	3
	Other genetic diagnosis*	8	4	3
	Total	9	6	6
**Cardiac/Renal N = 4 (3%)**	Primary Cardiac disease	1	1	1
	Renal Failure	1	0	0
	Total	2	1	1
***Immune Dysfunction N = 2 (2%)***	Immunodeficiency	1	1	0

*Other genetic diagnosis includes: Noonan syndrome, CFC syndrome, autoimmune polyendocrinopathy, deletion 1p36, 13q deletion, Coffin Lowry syndrome, congential disorder of glycosylation, Pierre Robin, Treacher Collins, Angelman, undiagnosed genetic syndrome

### Main Results

Univariate analysis was performed for the 4 HRQOL measures. Technology group, number of nursing hours and CSHCN screener score were the only 3 variables that were significantly different across all 4 HRQL scores (see Table 3).

**Table 3:** Univariate analysis for each of the HRQL instruments: HUI2, HUI3, CPCHILD, and PedsQL.

	Subcategory	HUI2	P-value	HUI3	P-value	CPCHILD	P-value	PedsQL	P-value
Group*			0.001		0.0001		0.0001		0.02
	G tube	0.4 (0.04)		0.1 (0.06)		57.7 (2.8)		46.9 (3.2)	
	LTHV+ GT	0.42 (0.05)		0.2 (0.08)		57.5 (3.5)		42.4 (4.1)	
	LTHV	0.7 (0.04)		0.5 (0.06)		77.2 (2.7)		56.2 (3.1)	
Gender*			0.1		0.07		0.2		0.4
	Male	0.5 (0.04)		0.2 (0.06)		62.9 (2.6)		47.8 (2.8)	
	Female	0.6 (0.04)		0.4 (0.06)		68.0 (2.7)		51.3 (2.9)	
Nursing Support*			0.0001		0.0001		0.0001		0.0001
	<5 hrs/wk	0.6 (0.03)		0.4 (0.05)		74.0 (2.1)		56.2 (2.4)	
	≥5 hrs/wk	0.3 (0.04)		0.03 (0.06)		51.7 (2.6)		38.5 (3.0)	
Female Caregiver Education* High School Post-Secondary			0.9		0.4		0.6		0.09
	High School	0.5 (0.05)		0.2 (0.08)		67.0 (3.7)		55.3 (3.9)	
	Post-secondary	0.5 (0.03)		0.3 (0.05)		64.8 (2.3)			
Family Annual Income*			0.2		0.23		0.06		0.2
	≤ $50 000	0.5 (0.05)		0.2 (0.07)		60.3 (3.1)		47.1 (3.3)	
	$50 000-$100 000	0.56 (0.04)		0.4 (0.06)		69.1 (2.5)		53.2 (2.7)	
	> $100 000	0.2 (0.3)		-0.1 (0.5)		44.6 (20.1)		23.9 (21.8)	
Ethnicity*			0.6		0.9		0.3		0.5
	Caucasian	0.5 (0.04)		0.3 (0.05)		67.1 (2.4)		50.5 (2.6)	
	Non-Caucasian	0.5 (0.05)		0.3 (0.07)		62.7 (3.1)		47.9 (3.2)	
Time on Technology *(yrs)		-0.002 (0.008)	0.8	-0.007 (0.01)	0.5	0.7 (0.6)	0.2	1.1 (0.6)	0.06
Length of Hospital* Admittance in the Last Year (days)		-0.003 (0.002)	0.09	-0.004 (0.002)	0.1	-0.2 (0.1)	0.1	-0.1 (0.1)	0.28
CHSCN Screener Score* (out of 14)		-0.05 (0.006)	0.0001	-0.06 (0.009)	0.0001	-2.9 (0.4)	0.0001	-2.9 (0.4)	0.0001
Number of Siblings*		0.02 (0.02)	0.5	0.01 (0.03	0.7	-0.2 (1.5)	0.9	-0.3 (1.6)	0.9

*Results presented as mean(SE)

Multiple regression models were generated for each HRQL measures using variables with p values <0.2 in the univariate analysis. Table 4 demonstrates the multiple regression analysis for the HUI2 and HUI3 scores.

**Table 4:** Mean scores (SE) for the HUI2 and HUI3 multiple regression models for variables with p-values determined using the F-test.

		HUI2		HUI3	
	Subcategory	Least Squares Mean	P-value	Least Squares Mean	P-value
**Group**			0.021	0.15 (0.06) 0.28 (0.07) 0.36 (0.06)	0.04
	G tube	0.42 (0.04)		0.15 (0.06	
	LTHV+ GT	0.5 (0.05)		0.28 (0.07)	
	LTHV	0.57 (0.04)		0.36 (0.06)	
**Nursing Hours**			0.008		0.02
	<5 hrs/wk	0.57 (0.03)		0.36 (0.05)	
	≥5 hrs/wk	0.42 (0.04)		0.17 (0.06)	
**CSHCN Screener**		-0.036 (0.006)	<0.001	-0.5 (0.01)	<0.0001
**Gender**		-	-		0.03
	Male	-	-	0.19 (0.05)	
	Female	-	-	0.34 (0.05)	

**Fig 1:**
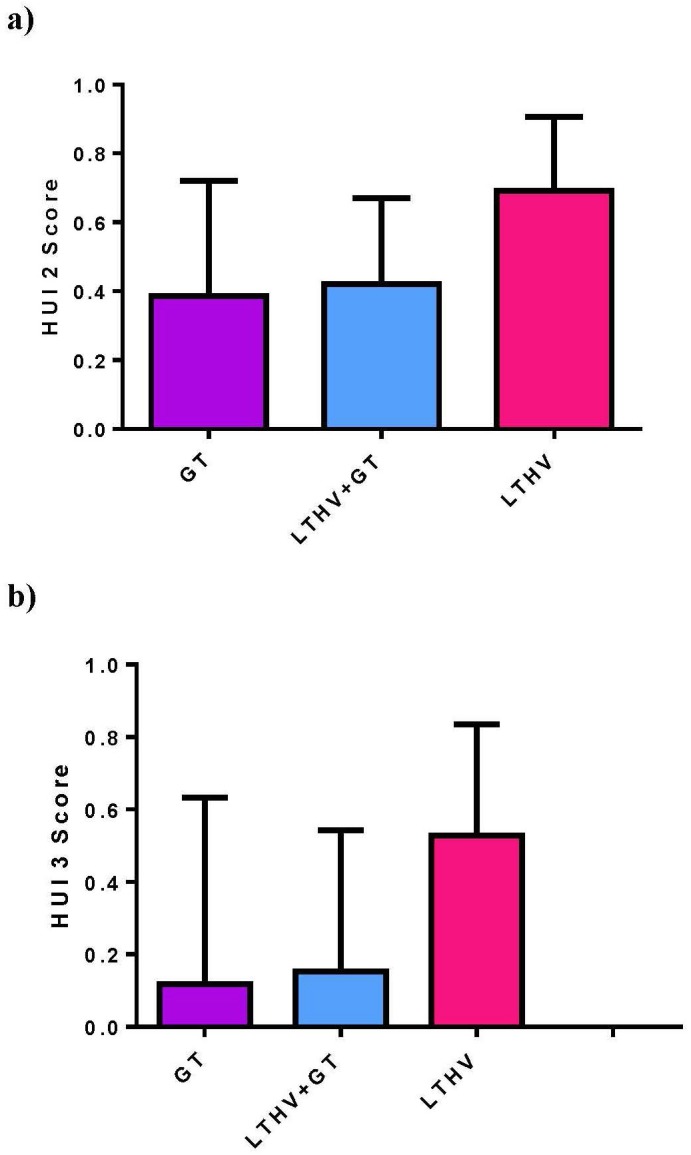
a) Mean (SD) HUI 2 scores for the GT, LTHV+GT and LTHV cohorts. b) Mean(SD) HUI3 scores for the GT, LTHV+GT and LTHV cohorts.

Group, number of nursing hours and CSHCN screener scores were significant predictors of HRQL as measured by the HUI2 and HUI3. The mean (SE) HUI2 score of the GT cohort, 0.42 (0.04) was significantly lower than the LTHV+GT cohort, 0.5 (0.05) and the LTHV cohort, 0.57 (0.04), P = 0.021 (see Fig 1A). Similarly in the HUI3 model, the GT cohort had a significantly lower mean (SE) score, 0.15 (0.06) compared to the LTHV+GT, 0.28 (0.07) and the LTHV cohort, 0.36 (0.06), P = 0.04 (see Fig 1B). Mean HUI2 and HUI3 scores were higher for children with <5 hrs/wk of nursing support than children with ≥5 hrs/wk. An increase in CSHCN screener mean (SE) score was correlated with decrease in HRQL as measured by the HUI2, -0.036 (0.006) as well as HUI3, -0.05 (0.01), P < 0.0001. Gender was also a significant predictor in the HUI3 regression analysis: females had a higher mean (SE) score than males, 0.34 (0.05) vs 0.19 (0.05), P = 0.03.

For the CPCHILD, the scores trended towards significance between the three cohorts for technology type only (p = 0.06). HRQL as measured by the Peds QL was not different across cohorts.

Single attribute analysis for the HUI2 and HUI3 scores was performed to further delineate the differences in the overall HUI2 and HUI3 scores between cohorts (see Table 5). In the HUI2 single attribute analysis sensation, mobility, cognition, and self-care were lower in those that had an enterostomy as compared to the LTHV cohort alone. Similarly, single attribute scores for HUI3 for vision, speech, ambulation, dexterity, cognition, and pain were lowest in the GT group or LTHV+GT cohort and highest in the LTHV group (p = 0.0001).

**Table 5:** HUI2 and HUI 3 single attribute results by cohort. Mean scores (SE) are presented.

	Gtube (n = 44)	LTHV+Gtube (n = 28)	LTHV (n = 47)	P-value
	**Health Utilities Index 2 Single Attribute Scores**			
Sensation	0.4 (0.05)	0.6 (0.07)	0.9 (0.05)	<0.0001
Mobility	0.4 (0.06)	0.4 (0.07)	0.7 (0.05)	<0.0001
Emotion	0.9(0.01)	0.9 (0.02)	0.9 (0.01)	0.7
Cognition	0.4 (0.06)	0.7 (0.07)	0.9 (0.06)	<0.0001
Self-care	0.2 (0.07)	0.2 (0.08)	0.6 (0.06)	<0.0001
Pain	0.9 (0.02)	0.9 (0.03)	0.9 (0.02)	0.09
	**Health Utilities Index 3 Single Attribute Scores**			
Vision	0.7 (0.05)	0.8 (0.06)	0.9 (0.04)	0.004
Hearing	0.9 (0.03)	0.9 (0.04)	0.9 (0.03)	0.9
Speech	0.4 (0.05)	0.6 (0.07)	0.9 (0.05)	<0.0001
Ambulation	0.4 (0.06)	0.3 (0.08)	0.6 (0.06)	0.0004
Dexterity	0.4 (0.06)	0.4 (0.08)	0.8 (0.06)	<0.0001
Emotion	0.9 (0.02)	1.0(0.02)	0.9 (0.01)	0.3
Cognition	0.4 (0.06)	0.7 (0.07)	0.9 (0.06)	<0.0001
Pain	0.9 (0.02)	0.8(0.02)	0.9 (0.02)	0.04

## Discussion

We are reporting on the first pediatric study to compare the HRQL in children using two different medical technologies at home: LTHV and enterostomy tubes. A key finding in our study was that the parent-reported HRQL of children using either of these technologies was poor. The advantage of using a validated, utility measure of HRQL is the ability to compare our cohorts with healthy children as well as other disease populations. This is helpful when discussing the long-term outlook for these children with families. Importantly, our results indicate that parental perceptions of the HRQL of children with LTHV and/or enterostomy tubes is much lower than the published literature for healthy children and children with complex chronic conditions such as Cystic Fibrosis **[30,34,35,36,37,38,39,40,41]**. There is a wide range of both HUI2 and HUI3 scores amongst the LTHV and the LTHV+GT cohorts consistent with the diagnostic heterogeneity of the cohort of children using LTHV.

There were several limitations of our study. Firstly, we used generic rather than disease-specific questionnaires for HRQL. The study participants are diagnostically heterogeneous with many different chronic complex conditions and hence disease-specific questionnaires could not be used. As stated earlier, the similar consequences of the medical technology (e.g. functional impairment, reliance on trained caregivers, shortened life-span, etc.) made it appropriate to cohort these children. Secondly, we did not measure the child’s perceptions of their HRQL, preferring instead to use parent-proxy reports for the study because over 50% of our LTHV population had a cognitive impairment. In other published studies of children with neurodevelopmental disabilities, parent (and professional reports) tend to be poorer than child reports of HRQL [42]. There is a need to formally compare parent-proxy and child self-report perceptions of HRQL in a large cohort using validated instruments. Thirdly, the questionnaires were completed at one time point only. As a result we are only able to report our observed associations between parental perceptions of HRQL in our cohort and the technology types. A prospective study in which HRQL is assessed pre and post the initiation of technology is needed to determine if the lower HRQL in the enterostomy cohort is technology based or a product of the underlying medical condition.

Another important finding in our study is that the reported HRQL in children using LTHV was higher than that of the enterostomy group, which is contrary to our original hypothesis. We found that children using both LTHV and an enterostomy tube had HRQL that was intermediate between the GT cohort and the LTHV cohort alone. Therefore, the presence of LTHV and a G tube was not additive in terms of its effect on HRQL measures. These results, taken together with the GT cohort having the lowest HRQL scores, challenges the traditional hierarchy of the burden associated with technology use at home. Evans et al reported that 75% of caregivers of children with enterostomy tubes at home report sleep disturbances even 1 year after tube insertion [43]. Therefore, an enterostomy tube, which has been traditionally thought of as minor technology dependence at home may in fact be more of a burden than previously appreciated.

Another potential explanation is that in our study, the children using enterostomy tubes were medically more complex with more severe neurodisability (lower cognition scores) thus explaining the lower parent proxy reported HRQL. Children in the GT cohort also had most severe needs of the three cohorts as reflected by their CSHCN scores. The CSHCN focuses on the needs consequences of chronic conditions with regards to functional limitation, services use and dependency. Therefore, higher CSHCN scores as compared to the LTHV and the LTHV+GT cohorts suggests that the GT cohort had higher levels of functional limitations, services usage and dependency that may be driving the lower HRQL scores. Furthermore, these children in the GT cohort had higher numbers of home nursing hours which is consistent with the higher CSHCN scores. Additionally, our LTHV cohort may also have been a relatively higher functioning group because the children were predominantly noninvasively ventilated. Only 10 (13%) of the LTHV cohort in our population were invasively ventilated. These numbers are consistent with the recent review of our home ventilation population in which we found that approximately 17% of our home ventilation program is invasively ventilated and the remainder were noninvasively ventilated [20]. Interestingly, post hoc comparison of HUI2 and HUI3 scores for the invasively and noninvasively ventilated study participants was not significantly different.

Previous research suggests that the insertion of an enterostomy tube does have a positive impact on health status, although caregivers continue to express several negative themes including intense caregiver needs, financial strain and social isolation [44]. Combining these results with our study, we can conclude that although insertion of an enterostomy tube can improve health status, the cohort in general have a low reported HRQL. Therefore, because a child’s HRQL relates to their physical, emotional and social well-being in addition to just their health status, it is important for clinicians to ensure children and their caregivers have the adequate social supports and are fully informed of the ongoing intense caregiving needs prior to the initiation of the medical technology. Our results may not be generalizable because this was a single center study. Our institution has the largest home ventilation program in the country and inserts all of the enterostomy tubes in our catchment area. Therefore, the clinics that the patients were recruited from were intended to represent a random sample of eligible patients for the study. However, there is a need for multicenter, national and international studies to better understand and characterize this medically complex population.

## Conclusions

In summary, we have described the parental perceptions’ of HRQL of children using two different technologies at home. The HRQL of both technology cohorts of children is relatively low as compared to healthy children and other disease cohorts. Additionally, we demonstrated that the HRQL of the LTHV was higher than children using enterostomy tubes, which is in contrast to our initial hypothesis. We have identified some factors that appear to be related to HRQL including nursing hours and CHSCN scores. Future studies are needed to determine the effect of LTHV initiation and enterostomy tube insertion on HRQL in larger cohorts aimed at identifying modifiable factors.

## Abbreviations

CNS: Central Nervous System
CPCHILD: Caregivers Priorities Caregiver Health Index
CSHCN: The Children with Special Health Care Needs
GT: Enterotomy Tube
HRQL: Health Related Quality of Life
HUI2/3: Health Utilities Index
LTHV: Long-Term Mechanical Ventilation at home
MSK: Musculoskeletal
NiPPV: Noninvasive Positive Pressure Ventilation
Peds QL: Paediatric Quality of Life Inventory
SD: Standard Deviation
